# Cross-species microbial genome transfer: a Review

**DOI:** 10.3389/fbioe.2023.1183354

**Published:** 2023-05-04

**Authors:** Mei-Chen Zhu, You-Zhi Cui, Jun-Yi Wang, Hui Xu, Bing-Zhi Li, Ying-Jin Yuan

**Affiliations:** Frontiers Science Center for Synthetic Biology and Key Laboratory of Systems Bioengineering (Ministry of Education), School of Chemical Engineering and Technology, Tianjin University, Tianjin, China

**Keywords:** synthetic genomics, microorganisms, genome transfer, cross-species, host platform

## Abstract

Synthetic biology combines the disciplines of biology, chemistry, information science, and engineering, and has multiple applications in biomedicine, bioenergy, environmental studies, and other fields. Synthetic genomics is an important area of synthetic biology, and mainly includes genome design, synthesis, assembly, and transfer. Genome transfer technology has played an enormous role in the development of synthetic genomics, allowing the transfer of natural or synthetic genomes into cellular environments where the genome can be easily modified. A more comprehensive understanding of genome transfer technology can help to extend its applications to other microorganisms. Here, we summarize the three host platforms for microbial genome transfer, review the recent advances that have been made in genome transfer technology, and discuss the obstacles and prospects for the development of genome transfer.

## 1 Introduction

Synthetic biology is an area that emerged in the early 21st century, and is based on elucidating and simulating the basic laws of biosynthesis (Kiga and Yamamura, 2008; Zhang et al., 2023). Its main application is the artificial design and construction of new biological systems, such as the establishment of bio-manufacturing pathways for drugs, functional materials, and energy substitutes (Bibi and Ahmed, 2020; Clarke and Kitney, 2020; Burgos-Morales et al., 2021). Genome synthesis is an essential part of this. It enables us to create living cells with fully controllable biological properties by *de novo* synthesis and assembly of rationally designed genomes (Baby et al., 2019; Labroussaa et al., 2019; Venetz et al., 2019; Zhang et al., 2020; Venter et al., 2022). Advances in synthetic genomics have facilitated the development of new tools and methods for synthesizing, assembling, modifying, and transferring complete microbial genomes (Lu et al., 2018; Liu et al., 2019). However, due to the slow growth rate, insufficient DNA recombination ability, and low transformation efficiency of the organisms, the tools and methods cannot always be performed directly in the original species. Therefore, it is necessary to transfer the genome into model organisms such as *Saccharomyces cerevisiae*, *Escherichia coli*, or *Bacillus subtilis* (Blount, 2015; Nielsen, 2019; Errington and Aart, 2020; Koster et al., 2022; Malci et al., 2022). Model organisms have the advantages of a short life cycle, a clear genetic background, easy cultivation, and a simple experimental procedure, making them very suitable as a platform for genome synthesis. The increasing size of artificially synthesized genomes and species poses a challenge not only for large genome synthesis, but also for genome transfer.

Whole genome transfer is the direct way to obtain complete genomes or eukaryotic chromosomes in other species, but in the case of unsuccessful genome transfer, a stepwise method can also be used to transfer the target genome (Karas et al., 2015). Genome transfer is divided into whole genome transfer and genomic fragment transfer. Whole genome transfer usually involves direct transfer into recipient cells (Karas et al., 2013a; Karas et al., 2014; Baby et al., 2018). For example, the whole genome of *Mycoplasma mycoides* was transferred into the related species *Mycoplasma capricolum,* thereby transforming one species into another (Lartigue et al., 2007). In contrast, genomic fragment transfer usually requires model organisms to act as platforms (Gibson et al., 2008b). A whole genome transfer process is divided into three parts: the first part involves the transfer of the entire genome or large genomic fragments into a suitable model organism. The second part is editing and modification, which is carried out using well-established genetic systems in the model organism. The final part is the transfer of the manipulated genome into the recipient cells of interest. The combination of genome transfer technology and genome engineering of model organisms is a powerful approach for manipulating both synthetic and natural microbial genomes (Gibson et al., 2010). If genome transfer technology can be applied to more microbial species, it could revolutionize microbial genetics and produce a new generation of artificially designed microorganisms.

In recent years, whole genome assembly technologies have flourished, with genome transfer playing an important role in this. With this aim in mind, this review sets out to describe the background of microbial genome transfer, especially cross-species transfer, focusing on the genome transfer using three different model organisms as platforms. In addition, we discuss the factors that influence genome transfer and examine its future prospects.

## 2 The background of cross-species microbial genome transfer

In 2005, it was demonstrated that whole genomes from other organisms could be transferred into *B. subtilis* (Itaya et al., 2005). The research involved transferring the *Synechocystis PCC6803* genome into the genome of *B. subtilis* cells, resulting in chimeric chromosomes. Subsequent research extended this approach to develop genome transfer methods using *B. subtilis* as a platform. Then, in 2007, Carole Lartigue et al. achieved the first complete genome transfer from *M. mycoides* to *M. capricolum* (Lartigue et al., 2007). In this experiment, the recipient genome was completely replaced by the donor genome. Based on this, the Venter research group achieved the complete chemical synthesis of the *Mycoplasma genitalium* genome in 2008 (Gibson et al., 2008a; Gibson et al., 2008b). Then, in 2010, the synthetic *M. mycoides* genome was transferred into *M. capricolum* cells, producing new *Mycoplasma* cells that could function normally (Gibson et al., 2010). The researchers used yeast as a temporary and modified platform for the synthetic genome (Gibson et al., 2008a; Gibson et al., 2008b). The cloning of the entire bacterial genome as centromeric plasmids in yeast was a breakthrough, allowing one-step genome transfer. Several extensions of this method have been developed in order to transfer whole prokaryotic genomes or eukaryotic chromosomes (Table 1). *Escherichia coli* is a commonly used model organism, and has the advantages of a short generation time, combined with simple and well-understood genetic manipulation methods (Ruiz and Silhavy Thomas, 2022). Although no studies have demonstrated the transfer of whole genomes into *E. coli*, megabase-sized plasmids can nonetheless be stably maintained in *E. coli* (Mukai et al., 2020). The development of methods to clone and maintain large genomic fragments in *E. coli* would greatly facilitate genome assembly and transfer technology. As mentioned above, both *B. subtilis* and *S. cerevisiae* are useful platforms for genome and chromosome transfer, and *E. coli* is also an important platform for maintaining the assembly of large DNA fragments.

**TABLE 1:** Summary of natural and synthetic genome transfer in yeast.

Source of genome	Prokaryotic or eukaryotic	Method	Cloned genome size (Mb)	G + C content (%)	References
*Mycoplasma genitalium*	Prokaryotic	Whole chromosome cloned in yeast	0.6	32	Benders et al. (2010)
*Mycoplasma hominis*	Prokaryotic	Whole chromosome cloned in yeast	0.665	27	Rideau et al. (2017)
*Mycoplasma putrefaciens*	Prokaryotic	Whole chromosome cloned in yeast	0.8	27	Labroussaa et al. (2016)
*Mycoplasma pneumoniae*	Prokaryotic	Whole chromosome cloned in yeast	0.8	40	Benders et al. (2010)
*Mesoplasma florum*	Prokaryotic	Whole chromosome cloned in yeast	0.8	27	Baby et al. (2018)
*Mycoplasma leachii*	Prokaryotic	Whole chromosome cloned in yeast	1.0	24	Labroussaa et al. (2016)
*Mycoplasma capricolum*	Prokaryotic	Whole chromosome cloned in yeast	1.1	24	Benders et al. (2010)
MGE-syn1.0 (Minimal Genome of *Escherichia coli*)	Prokaryotic	CasHRA	1.03	—	Zhou et al. (2016)
*Mycoplasma mycoides*	Prokaryotic	Whole chromosome cloned in yeast	1.1	24	Karas et al. (2019)
*Prochlorococcus marinus*	Prokaryotic	Whole chromosome cloned in yeast	1.66	36	Tagwerker et al. (2012)
*Spiroplasma citri*	Prokaryotic	Whole chromosome cloned in yeast	1.8	26	Labroussaa et al. (2016)
*Haemophilus influenza*	Prokaryotic	Whole chromosome cloned in yeast	1.8	38	Karas et al. (2013a)
*Chlamydomonas reinhardtii* (chloroplast genomes)	Eukaryotic	Whole chloroplast genome cloned in yeast	0.204	34	O'Neill et al. (2012)
*Phaeodactylum tricornutum*	Eukaryotic	Chromosomes 25 and 26	0.497/0.441	48	Karas et al. (2013b)
*Acholeplasma laidlawii*	Prokaryotic	Segment’s cloning and assembly in yeast	0.497/0.441	32	Karas et al. (2012)
*Synechococcus elongatus*	Prokaryotic	Segment’s cloning and assembly in yeast	0.454	55	(Noskov et al., 2012)

## 3 *Bacillus subtilis* platform for genome transfer

*Bacillus subtilis* is a typical platform used in many biotechnology and synthetic biology applications, and has proven itself to be an essential system for genome transfer (Johnston et al., 2014). *Bacillus subtilis* has the ability to take up exogenous DNA, and the exogenous DNA is usually integrated into the *B. subtilis* chromosome by RecA-mediated homologous recombination (Yadav et al., 2012; Yadav et al., 2014). Itaya et al. first proposed the use of the *B. subtilis* genome as a vector (BGM vector) for genomic sequence transfer (Itaya, 1995). They transferred a 48.5 kb length of *E. coli* prophage λ-DNA into *B. subtilis* by iterative assembly. The BGM vector is a new cloning and transfer system, and in order to test its ability to clone and transfer large genomic DNA, the same team cloned approximately 120 kb of mouse genomic DNA into BGM. The results showed that the stability of the mouse DNA could be maintained, proving that the BGM vector could at least carry DNA fragments up to 120 kb (Itaya et al., 2000; Itaya et al., 2003). Later, based on the BGM vector, the inchworm elongation method was proposed, in which the positioning and orientation of two DNAs will form an LPS (Landing Pad Sequences, LPS) array (LPA), and as the LPA slides, it leads to elongation of the adjacent target DNA (Itaya et al., 2005). To demonstrate the feasibility of this approach, the whole 3.5 Mb genome of *Synechocystis PCC6803* has been completely transferred into the *B. subtilis* genome. However, the inchworm extension method requires long, contiguous DNA as a template, which limits its application.

**FIGURE 1:**
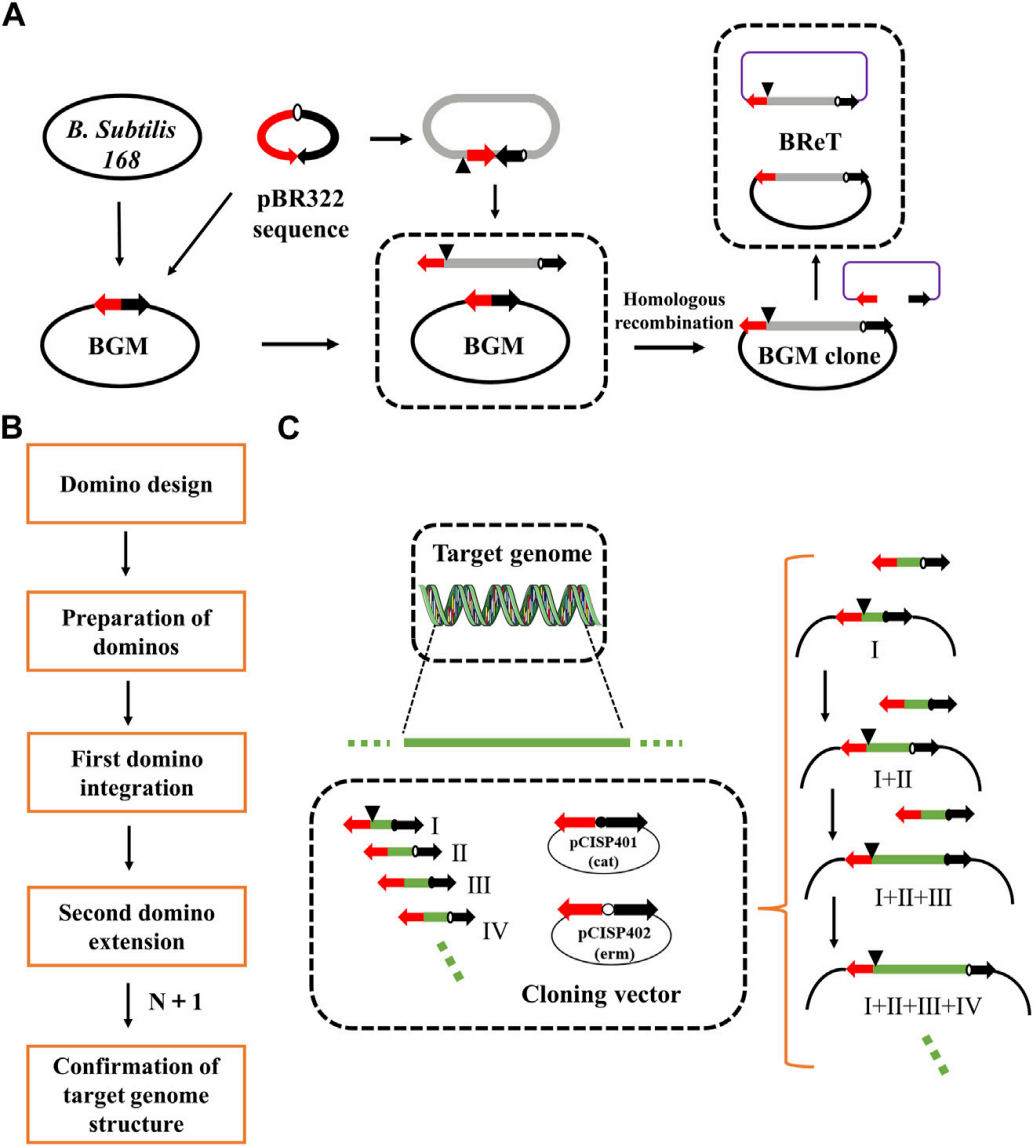
Domino cloning and BReT (*Bacillus* recombinational transfer retrieval). **(A)** Domino elongated DNA in the BGM vector by cloning between GpBR sequences, indicated by two arrows. BReT-mediated transfer occurs by homologous recombination between the pBR322 sequence of GpBR and the incoming linearized BReT plasmid; **(B)** the domino process; and **(C)** key steps of the domino process. DNA fragments are assembled into the BGM vector by homologous recombination between overlapping sequences. The dominoes were prepared in two plasmids, pCISP401 and pCISP402, with the alternating use of the two antibiotic selection markers allowing for multiple rounds of domino extension (cat, chloramphenicol; erm, erythromycin).

To overcome this limitation, Itaya et al. proposed the domino method, which connects DNA sequences in BGM vectors by homologous recombination between overlapping sequences to assemble large genomic fragments (Itaya et al., 2008) (Figure 1). The domino method has several advantages. For example, it does not require the preparation of large, high-purity DNA molecules, the structure of the final recombinant genome can be designed as desired, and the cloned DNA will maintain its structural stability. However, the domino method is usually used to transfer DNA smaller than 100 kb, and the transfer efficiency decreases significantly when the size of DNA increases to 100 kb. Therefore, researchers have developed a new conjugation transfer system that eliminates the domino method’s restrictions on the size of cloned DNA and thereby improves the transfer efficiency, achieving rapid transfer of 875 kb DNA (Itaya et al., 2018). Another study, aimed at simplifying the domino method, was conducted by Juhas et al., who combined Gibson Assembly and λ-red recombination in *E. coli* with RecA-mediated homologous recombination in *B. subtilis* (Juhas and Ajioka, 2016). The aim was to transfer bacterial artificial chromosome (BAC)-mediated DNA into the *B. subtilis* chromosome. Ultimately, they integrated the 10 kb DNA fragment from *E. coli* K12 MG1655 into the *B. subtilis* chromosome. To avoid irrational restructuring problems, Ogawa et al. developed an inducible recA expression BGM vector (iREX), which improved the stability of the inserted fragment by deleting endogenous recA and introducing a xylose-inducible recA expression cassette (Ogawa et al., 2015). Thus, the expression of recA was controlled by xylose in the medium.

## 4 Yeast platform for genome transfer

As a model organism, *S. cerevisiae* was the first eukaryote to be sequenced and has long been used as a platform to transfer DNA molecules from a variety of donor organisms in the form of yeast centromeric plasmids and yeast artificial chromosomes (Cherry et al., 2012; Vashee et al., 2020). Using yeast to transfer bacterial genomes or eukaryotic chromosomes requires the insertion of genetic elements from the yeast, including an autonomously replicating sequence (ARS), a centromere (CEN), and a selection marker to ensure that the cloned DNA can replicate and maintain itself. Genomes with low G + C% do not usually require an ARS, as the AT-rich consensus motif (ARS-like function) can occur naturally within their own sequence (Lartigue et al., 2009; Tan et al., 2021). To date, typical bacterial genomes and eukaryotic chromosomes have been transferred into yeast, with genome sizes ranging from 0.204 Mb to 1.8 Mb and GC content ranging from 24% to 55% (O'Neill et al., 2012; Karas et al., 2013a; Labroussaa et al., 2016).

**FIGURE 2:**
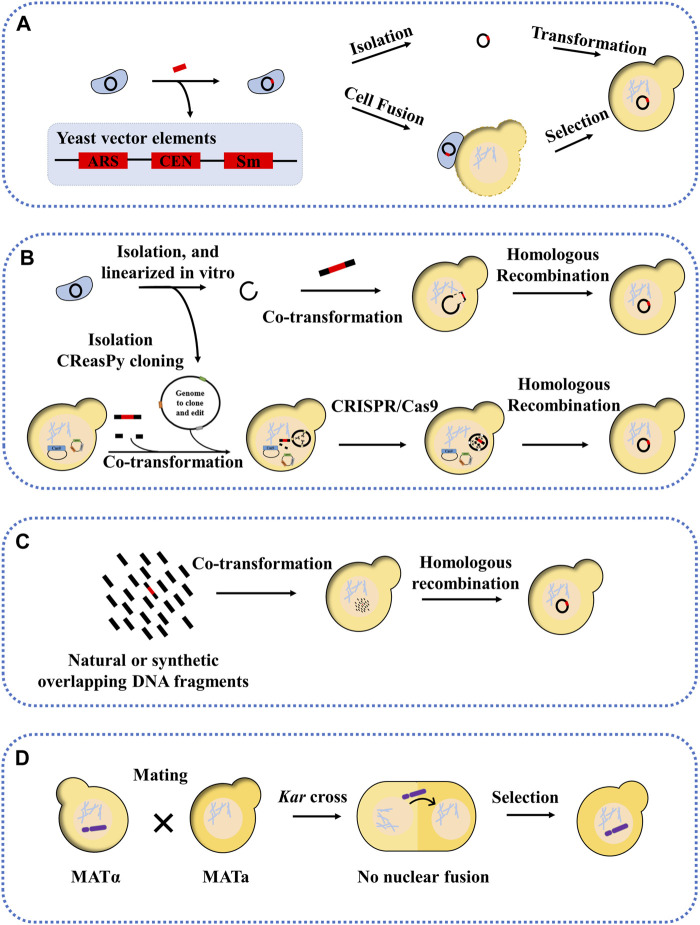
Methods for transferring natural or synthetic genomes into yeast. **(A)** Yeast sequence, necessary conditions for the propagation of foreign genomes in yeast (called the yeast vector), includes an autonomously replicating sequence (ARS), a centromere (CEN), a selection marker is inserted into the genome by transformation, after which the entire genome is isolated or cloned into the yeast by induced cell fusion; **(B)** the genome to be transferred is linearized and co-transformed into the yeast with the yeast vector, showing overlapping sequences and CReasPy cloning; **(C)** Cloning into yeast by assembling multiple overlapping fragments; **(D)** Kar cross transfer YAC.

The whole genome transfer into yeast can be carried out by means of centromeric plasmids. The first approach is to insert the yeast vector (ARS, CEN, and selection marker) into the genome prior to yeast transformation. After this, the newly marked genome can be transferred to yeast in two ways: one is completely isolated from donor cells and then transferred to the yeast, the other is transferred to the yeast by way of cell fusion (Figure 2A). Before cell fusion, cells need to be treated with enzymes, ultrasound, etc. to remove yeast cell walls and produce spheroplasted cells. Yeast spheroplasts can not only be transformed with purified DNA, but can also be fused with other yeast strains or bacterial cells to allow DNA transfer (Zhou et al., 2009; Benders et al., 2010; Tagwerker et al., 2012). The advantage of this method is that the yeast vector insertion site can be selected without affecting the viability of the donor cells. *Mycoplasma* have been successfully cloned in yeast, including *M. genitalium* (0.6 Mb), *Mycoplasma pneumoniae* (0.8 Mb), and *M. mycoides subspecies capri* (1.1 Mb) (Benders et al., 2010). These organisms were initially selected for genome cloning because of their small genome size and special genetic code (the UGA encoding tryptophan instead of a stop codon), which avoids toxicity to the host yeast cells. Subsequently, other bacterial genomes with standard genetic codes have also been successfully transferred into yeast, including the 1.8 Mb genome of *Haemophilus influenzae* and the 1.66 Mb genome of *cyanobacterium Prochlorococcus marinus MED4* (Tagwerker et al., 2012; Karas et al., 2013a). However, in another study, the genome of *Acholplasma laylawii PG-8A* failed to transfer into yeast using this method (Karas et al., 2012). The researchers found that a gene encoding an extracellular endonuclease was toxic to yeast*;* after inactivating this gene, its genome was found to be stable in yeast. The second approach is transformation-associated recombination (TAR) cloning, which exploits yeast’s ability to efficiently recombine DNA fragments (Lee et al., 2015). In this approach, the genome was isolated, then linearized *in vitro*, and finally co-transformed into yeast with a linear yeast vector containing homology sequences (Figure 2B) (Kouprina and Larionov, 2003; 2016; Rideau et al., 2017). For example, the genome of *Mycoplasma hominis* was transferred into yeast in a single step, and successfully modified using the CRISPR/Cas9 editing tool. A variation of this approach is the CReasPy cloning (Figure 2B). It combines CRISPR/Cas9 gene editing technology with the efficient homologous recombination of yeast to simultaneously transfer and edit the bacterial genomes. Using this approach, the 0.816 Mb genome of *M. pneumoniae* was successfully transferred into yeast (Ruiz et al., 2019). Another method is CasHRA, which combines CRISPR/Cas9, yeast homologous recombination, and yeast protoplast fusion (Zhou et al., 2016). This involves the co-introduction of multiple large circular DNAs into yeast by protoplast fusion, followed by linearisation by gRNA-guided Cas9 protein cleavage, and finally DNA assembly using the yeast homologous recombination system. Using CasHRA, Zhou et al. successfully assembled and transferred the 1.03 Mb minimal *E. coli* genome into yeast.

Genomic segment transfer methods, an extension of the TAR cloning technique, can be performed by using multiple overlapping fragments of yeast transformation (Figure 2C) (Gibson et al., 2008b; Karas et al., 2013b). For example, the 2.7 Mb genome of *Synechococcus elongatus* with 55% GC content was divided into 30 overlapping fragments with homologous arms, each approximately 112 kb in length. Similarly, for eukaryotic chromosomes, Karas et al. successfully assembled and transferred the chromosomes 25 and 26 of *Phaeodactylum tricornutum* in yeast using the TAR cloning technique, starting from a DNA fragment of approximately 100 kb. And in another study, the mitochondrial genome of *P. tricornutum* was also successfully cloned and transferred into yeast, with a size of 60 kb–95 kb (Cochrane et al., 2020). The above studies have shown that the transferred genomic fragments cannot exceed 200 kb, but the addition of ARS can significantly increase the transferred and assembled fragment length (454 kb) and increase the stability of larger genomic DNA in yeast.

The yeast artificial chromosome (YAC) is an efficient tool for transferring large genomic fragments (Coulson et al., 1988; Larin et al., 1991). Most YAC libraries are constructed in haploid yeast strains, and it is necessary to transfer the YAC from the host strain to the target yeast strain. Researchers have developed a new approach to efficiently transfer YAC into target yeast strains, a method known as *kar* cross (Figure 2D) (Spencer et al., 1994). This approach is based on the fact that yeast chromosomes can be transferred from one nucleus to another between *kar1* mutants and wild strains (Georgieva and Rothstein, 2002). The principle is based on the fact that, when yeast cells mate, nuclear fusion occurs immediately after cell fusion, with no intervening cell or nuclear division, resulting in a diploid. If the nuclear fusion gene (such as *kar1*, *kar2*, etc.) is mutated in one of the mating partners, nuclear fusion cannot occur, resulting in a heterokaryon containing two haploid nuclei (Dutcher, 1981; Yang and Kuang, 1996). In this case, it is possible that the target chromosome, such as YAC, could be transferred from one nucleus to the other (Torres et al., 2007). For example, Spencer et al. used a *kar1* mutant as a vector to transfer starch (*sta2*) and melibiose (*mel*)-utilizing genes into industrial strains of *S. cerevisiae* by single-chromosomal transfer (Spencer et al., 1992). In another study, Guo et al. used the *kar1* mutant approach; the four synthetic yeast chromosomes (synII, synV, synX, synXII) from the Synthetic Yeast Genome Project (Sc2.0) were transferred separately into wild-type yeast (Guo et al., 2022). In addition, Xu et al. used chromosome elimination via CRISPR-Cas9 to enable the chromosome transfer and demonstrated that chromosome XIV (chrXIV) is critical for the thermotolerance trait of the industrial strain Y12. In this study, the constructed heterozygous haploid, in which chrXIV from Y12 was transferred into BY4741 and the corresponding chrXIV of BY4741 was eliminated by CRISPR-Cas9, showed similar thermotolerance to the Y12 haploid. Through chromosome driving, the thermotolerance trait can be transferred into BY4741 (Xu et al., 2020).

## 5 *Escherichia coli* platform for genome transfer

*Escherichia coli* can maintain larger genomic fragments, which is also important for genome transfer. Here, we will mainly review *E. coli* as an assembly and transfer platform for genome or genomic fragments. There is a natural recombination system in *E. coli*, the RecA recombination system, which consists of the RecA and RecBCD proteins (Kowalczykowski, 2000). In practice, however, the RecA system has low recombination efficiency and requires a long homologous sequence (about 500 bp), which limits its application. Therefore, a more efficient *in vivo* recombination system has emerged in *E. coli* has emerged, λRed/ET, which relies on bacteriophage recombinases: either the Redα/Redβ recombinase from phage λ or the RecE/RecT recombinase from phage Rac (Zhang et al., 1998; Li et al., 2021). Redα and RecE are 5′-3′ ATP-independent nucleic acid exonucleases that can digest double-stranded DNA from the 5′end to the 3′end, exposing the 3′end of the DNA molecule, whereas Redβ and RecT are single-strand binding proteins with annealing and invasion functions. Redγ, another protein found in the λ phage, significantly enhances the recombination efficiency of Redα/Redβ. It was subsequently identified as an inhibitor of the RecB subunit of the RecBCD complex, preventing the degradation of linear DNA molecules by endogenous nucleases (Venkatesh and Radding, 1993; Paskvan et al., 2001; Murphy, 2007; Zhang et al., 2011). The λRed/ET technology can efficiently manipulate cloned genomes or genome-sized fragments (Yu et al., 2000). This technique was first used to construct a 43 kb gene cluster *myxochromide S* from *Stigmatella aurantiaca* in *E. coli* (Wenzel et al., 2005). Subsequently, biosynthetic gene clusters from other organisms have been constructed in *E. coli*, ranging in size from 11 kb to 106 kb (Wang et al., 2021). Previous recombination methods in *E. coli* relied on homologous recombination between linear and circular DNA molecules, which is less efficient (Zhang et al., 1998; Muyrers et al., 1999). However, the approaches using Redαβ or the truncated version of RecET are inefficient at mediating the homologous recombination between two linear DNA molecules. Therefore, Fu et al. used a full-length RecE/RecT, which significantly improved the recombination efficiency between two linear DNA molecules (Fu et al., 2012). In addition, genomic fragments can be cloned directly and transferred into *E. coli* by transformation. High-quality genomic sequences are obtained using low melting point agarose, ligated to vectors using enzymes, and then transformed to transfer genomic sequences into *E. coli*. Bacterial artificial chromosome (BAC) library construction technique is the traditional method for obtaining and transferring cross-species microbial genomic sequences into *E. coli*, but the method is time-consuming, labor-intensive, and the genomic sequences obtained are random. In recent years, many methods have emerged to obtain and transfer the targeted microbial genomic fragments into *E. coli*, such as CATCH, CAPTURE, ExoCET, TAPE, CAT-FISHING, etc. (Jiang et al., 2015; Wang et al., 2018; Enghiad et al., 2021; Cui et al., 2022; Liang et al., 2022). CATCH uses CRISPR/Cas9 technology to obtain the target genomic fragments and then uses Gibson assembly to clone the genomic fragments *in vitro*. Zhu et al. used this method to successfully transfer 150 kb of *E. coli* genomic sequence into *E. coli*. The CAPTURE method uses CRISPR/Cas12a to digest the genomic sequence and then uses a DNA assembly approach to obtain the genomic sequence. Zhao et al. obtained 113 kb of *Actinomycetes* genomic fragments in *E. coli* cells using this method. ExoCET combines nucleic acid exonuclease with the intracellular RecET protein of *E. coli* to synergistically obtain genomic DNA. Using this method, Zhang et al. acquired the 106 kb of genomic DNA from *S. albus DSM41398*. Li et al. developed the TAPE method, which uses the linear plasmid vector to target the genomic sequence, and they eventually transferred the 156 kb genomic sequence of *E. coli* into the *E. coli*. The CAT-FISHING technique used CRISPR/Cas12a to excise chromosome sequences and ligate them to the vector, Tan et al. captured 145 kb-long genomic DNA sequence from *S. albus J1074* in *E. coli* by this method. However, it is difficult to obtain high quality genomic sequences by using low melting point agarose gels, and the length of the transferred genome is also limited.

## 6 Conclusions and perspectives

**FIGURE 3:**
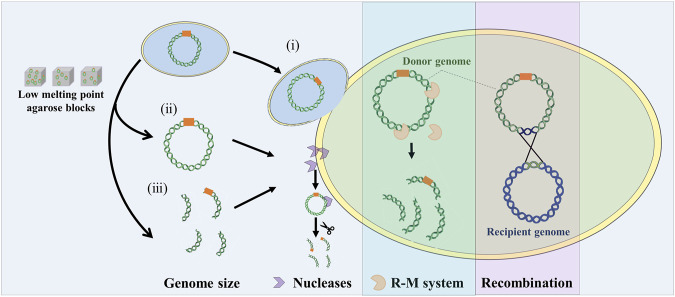
Influence factors on genome transfer. Whole genome transfer by cell fusion (i), or purified genomic DNA using low melt agarose block (ii). Genomic fragment transfer (iii). R-M system: restriction modification system. Recombination: two genomes can recombine to form a mosaic or hybrid genome.

The combination of genome engineering and genome transfer is a new approach for manipulating natural and synthetic genomes. Whole genomes and genomic segments in model organisms need to be transferred into recipient cells that are suitable for their expression. How to transfer the genome from the model organism into the final recipient cell is the difficult part of genome transfer technology. This requires the isolation and purification of large DNA *in vitro* or using cell fusion to transfer the donor genome into the recipient cell with the treatment of PEG. And due to genome size, phylogenetic distance, non-specific nucleases, restriction modification systems, and some unknown factors, the genome transfer is limited to a small set of *mycoplasma* species (Figure 3).

Firstly, genome size is one of the important factors limiting genome transfer. Usually, the genome is extracted for transfer, but large DNA molecules are susceptible to breakage by shearing forces. Extracting high quality, large volume, intact genomes using low melting point agarose gels requires delicate technical manipulation. To circumvent this problem, Karas et al. directly transferred the bacterial genome into the yeast by PEG induction under conditions that promote cell fusion (Karas et al., 2013a). Currently, *H. influenzae* (1.8 Mb) is reported to be the largest genome transferred into yeast, but it is unclear whether the larger bacterial genome can be transferred into yeast. By improving cell fusion methods, this problem may be overcome. In addition, genome size is closely related to GC content. Genomes with relatively high GC content require the insertion of additional ARS to be stably maintained in yeast cells.

Secondly, the effect of the phylogenetic distance between donor and recipient on genome transfer needs to be better understood. When the donor genome enters the recipient cell, the recipient cell must be able to transcribe and translate the genes of the donor genome until the genome can replicate, transcribe, and translate on its own (Lartigue et al., 2007; Labroussaa et al., 2019). The molecular mechanisms of the recipient cell and the donor genome must be compatible. Labroussaa et al. investigated the effect of evolutionary distance between donor and recipient species on the efficiency of genome transfer (Labroussaa et al., 2016). The results showed that the closer the genomes of the donor and recipient cells were to each other, the higher the transfer efficiency.

Thirdly, the non-specific nucleases. These can be secreted into the environment or bound to membranes, and can even cleave the donor genome, so they need to be inactivated before genome transfer (Sharma et al., 2015). For example, the *A. laylawii PG-8A* genome mentioned above was successfully cloned into yeast after knocking out the gene encoding the extracellular nucleic acid endonuclease (Karas et al., 2012).

Finally, the restriction modification system is a defense mechanism against foreign DNA invasion, and the donor genome is recognized as exogenous DNA by the recipient cells. When transferring other bacterial genomes from yeast, it may be necessary to methylate the donor genome *in vitro* to protect it from restriction enzymes in recipient cells. Karas et al. found that removing the restriction modification system from the *Mycoplasma mycoides* JCVI-syn1.0 genome in yeast increased the efficiency of genome retransfer (Karas et al., 2013a; Karas et al., 2019).

In addition, there are some species-specific factors. For example, when a donor genome enters a recipient cell, the two genomes can recombine to form a mosaic or hybrid genome. The cytoskeleton and the nuclear membrane are also important influencing factors. Brown et al. proposed to synchronize recipient cells into the M (mitotic) phase, a period when the nuclear membrane and cytoskeleton are in a state of remodeling. Experimental results have shown that membrane fusion transfer using mammalian cells synchronized to mitosis can be almost 300 times more efficient (Brown et al., 2017).

Based on the above, the tasks required to adapt genome transfer technologies to other species should include: (1) Finding new recipient cells that are phylogenetically closer to the donor genome. (2) Modifying the recipient cells: one example is the introduction of genes associated with transcription, translation, and replication of the donor genome into recipient cells prior to transfer. (3) Improving protocols for the preparation of recipient cells: synchronizing the recipient cells to the M phase (mitosis) during cell fusion.

## Abbreviations

*B. subtilis*, *Bacillus subtilis*; *M. mycoides*, *Mycoplasma* mycoides; *M. capricolum*, *Mycoplasma* capricolum; *E. coli, Escherichia coli*; *S. cerevisiae*, *Saccharomyces cerevisiae*; BGM, *B. subtilis* genome; LPS, Landing Pad Sequences; LPA, Landing Pad Sequences array; ARS, autonomously replicating sequence; CEN, centromere; *M. genitalium*, *Mycoplasma*. *Genitalium*; *M. pneumoniae*, *Mycoplasma*; *M. mycoides subspecies capri*, *Mycoplasma subspecies capri*; TAR, Transformation Associated Recombination; CRISPR/Cas9, Clustered Regularly Interspaced Short Palindromic Repeat; CReasPy cloning, a Method for Simultaneous Cloning and Engineering of Megabase-Sized Genomes in Yeast Using the CRISPR-Cas9 System; CasHRA, Cas9-facilitated Homologous Recombination Assembly; YAC, Yeast Artificial Chromosome; ATP, Adenosine-Triphosphate; BAC, Bacterial Artificial Chromosome; CATCH, Cas9-Assisted Targeting of Chromosome segments; CAPTURE, Cas12a-assisted Precise Targeted cloning Using *in vivo* Cre-lox Recombination; TAPE, the TelN/tos-assisted Precise Targeting of Chromosome segments; CAT-FISHING, CRISPR/Cas12a mediated fast direct biosynthetic gene cluster cloning; ExoCET, exonuclease *in vitro* assembly combined with RecET recombination; M phase, mitotic phase.
